# Efficacy and safety of basal insulins in people with type 2 diabetes mellitus: a systematic review and network meta-analysis of randomized clinical trials

**DOI:** 10.3389/fendo.2024.1286827

**Published:** 2024-03-21

**Authors:** Mohsen Dehghani, Masoumeh Sadeghi, Farzaneh Barzkar, Zohreh Maghsoomi, Leila Janani, Seyed Abbas Motevalian, Yoon K. Loke, Faramarz Ismail-Beigi, Hamid Reza Baradaran, Mohammad E. Khamseh

**Affiliations:** ^1^ Department of Epidemiology, School of Public Health, Iran University of Medical Sciences, Tehran, Iran; ^2^ Metabolic Syndrome Research Center, Mashhad University of Medical Sciences, Mashhad, Iran; ^3^ Department of Epidemiology, Faculty of Health, Mashhad University of Medical Sciences, Mashhad, Iran; ^4^ Endocrine Research Center, Institute of Endocrinology and Metabolism, Iran University of Medical Sciences, Tehran, Iran; ^5^ Research Center for Prevention of Cardiovascular Disease, Institute of Endocrinology and Metabolism, Iran University of Medical Sciences, Tehran, Iran; ^6^ Imperial Clinical Trials Unit, Imperial College London, London, United Kingdom; ^7^ Norwich Medical School, University of East Anglia, Norwich, United Kingdom; ^8^ Department of Medicine, Case Western Reserve University, Cleveland, OH, United States; ^9^ Ageing Clinical and Experimental Research Team, Institute of Applied Health Sciences, University of Aberdeen, Aberdeen, Scotland, United Kingdom

**Keywords:** basal insulin, blood glucose, body weight, diabetes treatment, hypoglycemia, network meta-analysis

## Abstract

**Aim:**

The comparative effectiveness of basal insulins has been examined in several studies. However, current treatment algorithms provide a list of options with no clear differentiation between different basal insulins as the optimal choice for initiation.

**Methods:**

A comprehensive search of MEDLINE, Embase, Cochrane Library, ISI, and Scopus, and a reference list of retrieved studies and reviews were performed up to November 2023. We identified phase III randomized controlled trials (RCTs) comparing the efficacy and safety of basal insulin regimens. The primary outcomes evaluated were HbA1c reduction, weight change, and hypoglycemic events. The revised Cochrane ROB-2 tool was used to assess the methodological quality of the included studies. A random-effects frequentist network meta-analysis was used to estimate the pooled weighted mean difference (WMD) and odds ratio (OR) with 95% confidence intervals considering the critical assumptions in the networks. The certainty of the evidence and confidence in the rankings was assessed using the GRADE minimally contextualized approach.

**Results:**

Of 20,817 retrieved studies, 44 RCTs (23,699 participants) were eligible for inclusion in our network meta-analysis. We found no significant difference among various basal insulins (including Neutral Protamine Hagedorn (NPH), ILPS, insulin glargine, detemir, and degludec) in reducing HbA1c. Insulin glargine, 300 U/mL (IGlar-300) was significantly associated with less weight gain (mean difference ranged from 2.9 kg to 4.1 kg) compared to other basal insulins, namely thrice-weekly insulin degludec (IDeg-3TW), insulin degludec, 100 U/mL (IDeg-100), insulin degludec, 200 U/mL (IDeg-200), NPH, and insulin detemir (IDet), but with low to very low certainty regarding most comparisons. IDeg-100, IDeg-200, IDet, and IGlar-300 were associated with significantly lower odds of overall, nocturnal, and severe hypoglycemic events than NPH and insulin lispro protamine (ILPS) (moderate to high certainty evidence). NPH was associated with the highest odds of overall and nocturnal hypoglycemia compared to others. Network meta-analysis models were robust, and findings were consistent in sensitivity analyses.

**Conclusion:**

The efficacy of various basal insulin regimens is comparable. However, they have different safety profiles. IGlar-300 may be the best choice when weight gain is a concern. In contrast, IDeg-100, IDeg-200, IDet, and IGlar-300 may be preferred when hypoglycemia is the primary concern.

## Introduction

Diabetes Mellitus (DM) is a chronic and progressive metabolic disorder that affects 9.3% of the world population (463 million people), with increasing incidence and prevalence (1). Type-2 diabetes mellitus (T2D) accounts for 90-95% of all cases and is caused by progressive insulin resistance and relative insulin deficiency (1). T2D can initially be treated with diet, increased physical activity, and oral glycemia-lowering medications (2, 3). However, as the disease progresses, many patients require and benefit from additional medications, including insulin, due to the progressive and gradual loss of insulin-producing cells in the pancreas over 5 to 10 years (4). Insulin initiation and intensification are required when lifestyle modifications, oral glucose-lowering drugs (OGLDs), Glucagon-like peptide 1 receptor agonists (GLP-1RAs), and SGLT2 inhibitors fail to provide adequate glycemic control (5, 6).

Although insulin was introduced nearly a century ago, and its use has prolonged survival and has saved the lives of countless people with diabetes, significant unmet needs remain (7–11). The proportion of people with T2D who achieve their glycemic goals with hypoglycemic events remains high (7, 9–11). This study focuses on various long-acting (basal) insulin preparations, their usage, efficacy, and side effects.

Neutral Protamine Hagedorn (NPH) insulin, a long-acting basal insulin preparation, has been considered the standard treatment for many years, and a large number of insulin analogs have been produced in recent years and are increasingly used in the treatment of diabetes (11). Despite the fundamental advances in their design, pharmacokinetics, and pharmacodynamics, hypoglycemia and weight gain remain two major problems with their use (8, 10). Significant improvements have been made with the introduction of long-acting insulin analogs regarding glycemic variability and risk of hypoglycemia (7, 11). Furthermore, new generations of basal insulin analogs have been developed to improve their efficacy and safety (11–15).

Several studies have compared first and second-generation basal insulins regarding glycemic control, weight gain, and hypoglycemic events; however, their results have been inconsistent and largely compared to insulin glargine and NPH (14–20). In the absence of adequate data on direct comparisons, network meta-analysis can synthesize evidence from direct and indirect comparisons of multiple interventions to determine the best available treatment option (21, 22).

The results of the present comprehensive systematic review and network meta-analysis address the challenging question regarding selecting the “best” treatment alternative (preferred or with a high priority to choose) among basal insulins. The purposes of the study included: determining the benefits (efficacy) and risks (safety) of basal insulins in T2D and determining the best treatment alternative (preferred or with a high priority to choose) among basal insulins in terms of glycemic control, weight gain, and hypoglycemic events. This can help clinicians, patients, and policymakers decide the best treatment options with optimal balance for increased efficacy and less harm.

## Methods

The PRISMA-NMA guideline was followed to report the present systematic review and network meta-analysis (23). The protocol was registered in the International Prospective Register of Systematic Reviews, PROSPERO (CRD42022325625).

### Search strategy

A comprehensive search of online databases, including MEDLINE, Embase, Cochrane Library, ISI, and Scopus, was performed through November 2023, using the MeSH terms ‘basal’ or ‘basal insulin’ or ‘neutral protamine Hagedorn’ or ‘NPH’ or ‘isophane’ or ‘insulin lispro protamine suspension’ or ‘ILPS’ or ‘human insulin’ or ‘glargine’ or ‘lantus’ or ‘long-acting insulin’ or ‘ultra long-acting insulin’ or ‘insulin analogue’ or ‘detemir’ or ‘levemir’ or ‘Toujeo’ or ‘degludec’ or ‘Tresiba’ AND ‘efficacy’ or ‘safety’ or ‘benefit’ or ‘risk’ or ‘glycemic’ or ‘glycated hemoglobin’ or ‘fasting plasma glucose’ or ‘fasting blood sugar’ or ‘body weight’ or ‘weigh gain’ or ‘hypoglycemia’ AND ‘diabetes mellitus.’ Also, meeting abstracts, ClinicalTrials.gov, and the annual meeting abstract books of the ADA and EASD were searched. The time and language of publications were not restricted. In addition, we contacted experts and other researchers in the field for ongoing studies and additional data using Email. We reviewed the reference lists of retrieved publications and relevant reviews for further pertinent studies. Duplicate publications were removed. Two authors (MD and MS) independently screened the studies based on titles and abstracts, and finally, the full texts were reviewed in case the studies met the inclusion criteria based on the title and abstract. Conflicts were resolved by consensus.

### Selection criteria

All phase III randomized controlled trials (RCTs) that compared basal insulins (long and ultra-long-acting insulins) with each other for study outcomes (glycemic control, weight gain, and hypoglycemia) in adults with T2D were included in the present systematic review. The population of interest was defined as adults with T2D, including those who were newly initiating insulin (insulin-naïve and on OGLDs), as well as individuals who were already exposed to insulin. This encompassed a diverse range of patients, reflecting both those at the initiation stage of insulin therapy and those with prior exposure to insulin treatments. Our decision to include both subgroups is supported by the findings of Freemantle et al. (24), who performed subgroup analysis specifically on studies assessing outcomes in insulin-naïve patients. Remarkably, their results indicated that the estimates remained largely unchanged even after including all studies, irrespective of the pretreatment status. Regarding the assessed interventions among RCTs, studies were considered eligible as long as the treatment regimens of the study arms contained at least two distinct basal insulins, including all intermediate/long and ultra-long-acting basal insulins including NPH, ILPS, glargine, 100 U/mL (IGlar-100); glargine, 300 U/mL (IGlar-300); detemir (IDet); degludec, 100 U/mL (IDeg-100); degludec, 200 U/mL (IDeg-200); and thrice-weekly degludec (IDeg-3TW). When the interventions comprised basal-bolus insulin regiments, the RCTs were included only when the short-acting components of the treatment regimens in various arms were precisely the same. Studies on Premixed insulin preparations and insulin peglispro were excluded (25). HbA1C, FPG, Body weight, Overall, Nocturnal, and severe hypoglycemia were all considered primary outcomes of the present study. RCTs were included when they assessed and compared these outcomes in T2D patients between different intervention arms. A minimum follow-up time of 12 weeks was also deemed an inclusion criterion. Inhaled insulins were also excluded. Glargine biosimilars (including MK-1293, MYL-1501D, LY2963016, etc.) were also excluded due to their similar efficacy and safety profile and similar pharmacokinetic and pharmacodynamics properties with the reference product (glargine). An adequate and standard randomization process in the RCTs was considered a vital inclusion criterion. The studies were included in the review if the two treatment arms were similar regarding main baseline variables, namely diabetes duration, mean age of the participants, HbA1c level, and body mass index. In addition, patients’ antidiabetic treatments prior to study initiation were assessed. In studies evaluating patients with different treatment backgrounds, ensuring that the two arms did not differ regarding the pretreatment medications or that pretreatments were considered during the randomization and stratification of patients was crucial.

**Figure 1:**
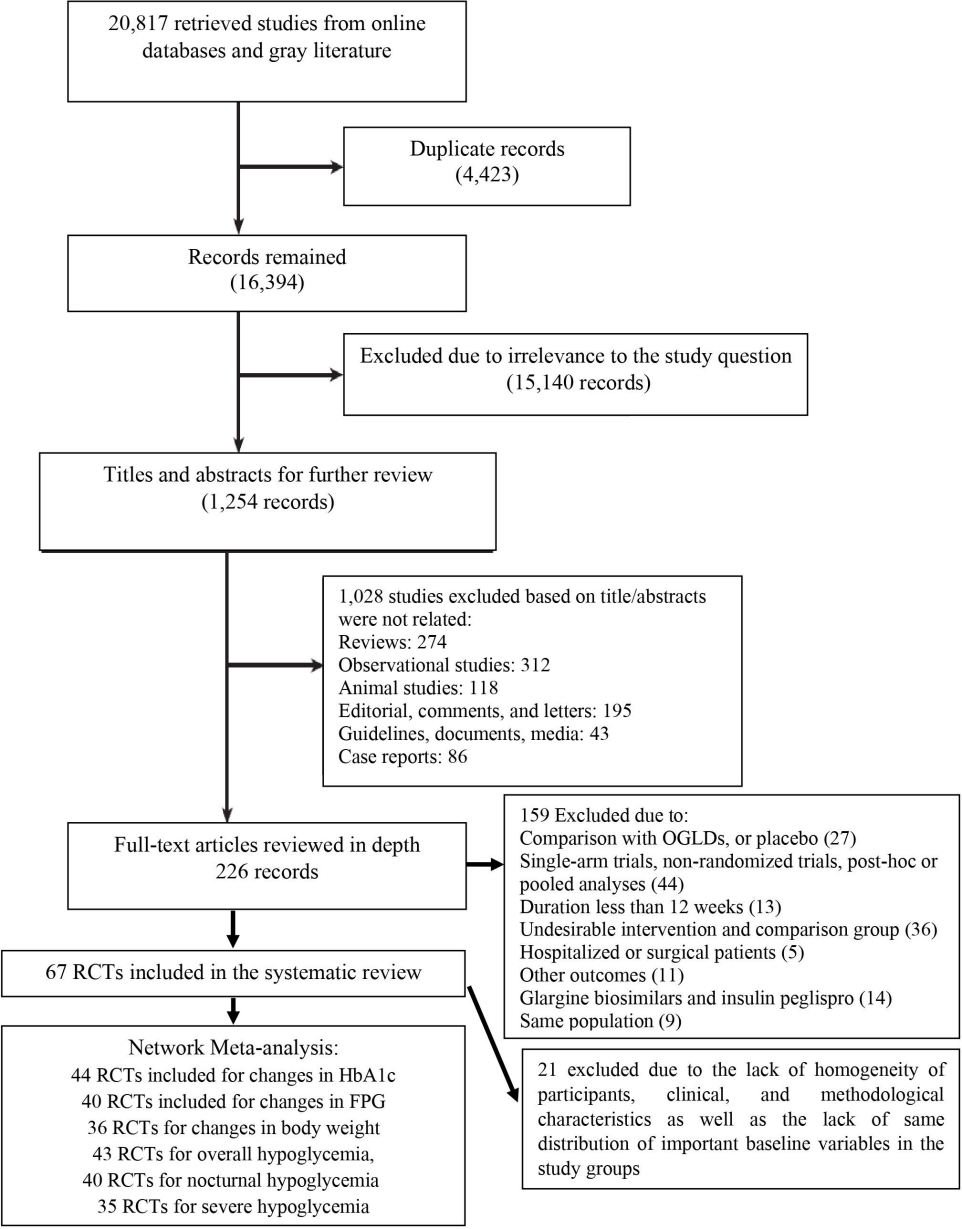
Flowchart for selection of studies.

The following studies were excluded: those with no control group, non-randomized trials, *post-hoc* or pooled analyses, real-world data, OGLDs/placebo/unknown comparison groups, unknown treatment period, follow-up period less than 12 weeks, data registries, non-standard randomized clinical trial, incorrect, insufficient or incomplete statistical analyses. In addition, studies including participants with cardiovascular or renal diseases or people with known risk factors for cardiovascular diseases were excluded. **Figure 1** shows the process of identifying, screening, and selecting randomized controlled trials to include in the systematic review and final analyses.

### Data extraction and risk of bias assessment

The main characteristics that were extracted from eligible studies were as follows: author name, publication year, name of the country, gender, age, body weight, body mass index, fasting blood sugar, HbA1c, study setting and population, duration of follow-up, type of diabetes, diabetes duration, number of patients in each treatment group, treatment regimens, insulin dosage, study outcomes (change in HbA1c from baseline, change in fasting plasma glucose (FPG) from baseline, change in body weight from baseline, the number of patients experiencing any/overall, nocturnal, or severe hypoglycemia events, effect size estimates (odds ratio or mean difference) and their 95% confidence intervals. Two reviewers (MD and MS) extracted the data independently, and the observed differences were resolved by consensus of review team members.

To assess the risk of bias or methodological quality of included RCTs, the new and revised Cochrane risk of bias tool (ROB-2) was used (26). The tool consists of five domains: bias based on the randomization process, deviations from intended interventions, missing outcome data, measurement of the outcome, and selection of the reported results. Each domain contains several questions, and the answer to each question has five options (yes, probably yes, probably no, no, no information). At the end of each domain, a risk of bias judgment is made for the domain as either low risk of bias, some concerns, or high risk of bias. Finally, an overall judgment of the risk of bias is made for each study, which summarizes all five domains (26). Two reviewers (MD and MS) assessed the risk of bias in studies, and three (HRB, LJ, and FB) verified it.

### Data synthesis and analysis

Basal insulin regimens were compared in all RCTs that were included. Treatment effects were estimated as mean differences (MDs) with 95% confidence intervals in HbA1c, fasting plasma glucose, and body weight (continuous outcomes), or odds ratios (ORs) with 95% confidence intervals (CIs) for any/overall, nocturnal, and severe hypoglycemia. Random-effects network meta-analyses within frequentist general linear mixed model framework were performed for data analyses. Network plots containing all basal insulin treatments were drawn for each outcome (27).

Extended forest plots were used to show pairwise comparisons of treatments using direct evidence. To assess the uncertainty in the estimated treatment effects, which includes the extent of heterogeneity, the predictive interval (PrI) plot was also used. Estimated treatment effects in a random-effects network meta-analysis for pairwise comparisons of all evidence in the network (both direct and indirect evidence) with 95% CIs were displayed using a league table. Diagonal cells contain the names of competing treatments in the network in the league table.

The assumption of heterogeneity was examined both qualitatively and quantitatively using study and participant characteristics across all eligible trials and the magnitude of tau2 (between study variance). We assessed the transitivity assumption by considering the distributions of potential effect modifiers (age, diabetes duration, baseline HbA1c level, and baseline body mass index) across pairwise comparisons. A design-by-treatment approach was used to check the consistency assumption in the entire analytical network (28). A loop-specific approach was applied to evaluate the presence of inconsistency locally in each closed loop (29). Inconsistency factor (IF) for continuous outcomes and the ratio of two odds ratios (ROR) for dichotomous outcomes in each closed loop and the ifplot was used for this purpose. The node-splitting method was also used to assess the inconsistency of the model by separating evidence on particular comparisons into direct and indirect evidence (30).

Sensitivity analyses were conducted by restricting the analyses to studies with a sample size of more than 100 participants in each treatment arm of the trial, only trials that had a low risk of bias, and trials with a parallel design (excluding the crossover designs) to assess the generalizability and the robustness of the network meta-analyses findings for each outcome. If any differences were observed in the distribution of effect modifiers (participants’ baseline characteristics) across the studies, subgroup analyses and, in particular, meta-regression were performed. The results before and after the adjustment with the effect modifiers were evaluated to derive clinically valid conclusions.

To rank the basal insulins for an outcome, we used mean rank and surface under the cumulative ranking (SUCRA) cumulative probabilities that express (as a percentage) the efficacy or safety of every intervention relative to an imaginary intervention that is always the best without uncertainty. Consequently, larger SUCRA scores might indicate a more effective or safer intervention. The presence of small-study effects in a network meta-analysis was assessed by comparison-adjusted funnel plots (31). All network meta-analyses were performed using Stata version 17.0 (Stata Corp., College Station, TX, USA) with a multivariate meta-analysis model using the mvmeta command and routines in Stata and R version 3.3.0 using the netmeta package.

### Assessing certainty of the evidence

We used the approach suggested by the GRADE (Grading of Recommendations Assessment, Development, and Evaluation) working group for rating the certainty of evidence for each network estimate (32–35). This approach ranks the overall certainty in effect estimates from very low to high (4 levels).

We initially rated the certainty of direct estimates for each pair-wise comparison. At this stage, since all the evidence was from RCTs, we started at high certainty in effect estimates and rated down in cases of serious concerns regarding the risk of bias, publication bias, indirectness, and inconsistency. Then, we rated the certainty in indirect estimates, focusing on the dominant lowest-order loop that contributed to the indirect estimate. The indirect estimate would be further rated down for concerns regarding intransitivity. The main effect-modifying factors that were considered to cause conceptual transitivity were baseline BMI, baseline HbA1c, duration of follow-up, and duration of DM. Cut-offs for decision-making were set based on the standard minimally clinically important differences as suggested by previous studies in patients with T2D.

To judge the overall certainty in the network estimates, we selected the certainty in the estimate that contributed more to the network estimate between the direct and indirect estimates as a baseline. We then considered rating down the certainty of the network estimate if there was incoherence (local inconsistency) between the contributing direct and indirect estimates. The certainty of the network estimate was subsequently assessed for serious or very serious imprecision that would further downgrade the overall certainty in the effect estimate according to the GRADE guidance articles (33, 34).

Using a minimally contextualized approach, we then chose the intervention with the highest number of studies (IGlar-100) as the reference and categorized the interventions for each outcome into three colored groups relative to this reference: 1) Among the most effective/least harmful (Green), 2) Not convincingly different from standard treatment (Yellow), and 3) Among the most harmful/least effective (Red). We further categorized each of these groups based on certainty about the effect estimate of the intervention relative to IGlar-100 as obtained from the GRADE approach, coloring the “very low” to “low” certainty evidence with light colors and “moderate” to “high” certainty evidence in dark colors (36, 37).

## Results

### Characteristics of RCTs

We identified 20,817 records through a comprehensive systematic search of databases. One thousand two hundred-and-thirty-three (1,254) records remained after removing duplicates and studies unrelated to the research question. After excluding the studies that did not meet the eligibility criteria, 226 full texts were assessed and selected 67 RCTs in the systematic review based on the defined criteria. In our network meta-analyses, we conducted separate analyses for changes in HbA1c (44 RCTs included), changes in body weight (38 RCTs included), overall hypoglycemia (43 RCTs included), and nocturnal hypoglycemia (40 RCTs included). Inclusion criteria were based on methodological, clinical, and baseline homogeneity, as illustrated in **Figure 1**.

**Supplementary Table 1** demonstrates the main characteristics of the 44 randomized controlled clinical trials included in this analysis (14, 17, 18, 38–78). The included trials were published between 2000 and 2021. Most trials were multicenter and multinational from most to all continents and with relatively large sample sizes. Only five studies had a total sample size of smaller than 100 participants, i.e., less than 50 patients in each group (31, 45, 61, 70, 71), and about 80% of trials had included more than 100 patients in each study arm (**Supplementary Table 1**). Three studies were conducted as a crossover clinical trial design (39, 42, 61), and the rest were parallel clinical trials. We handled these crossover trials similarly to parallel trials by only extracting data from the first period (79). Regarding the sample size and design of trials, sensitivity analyses were performed to assess the robustness of the findings.

Both men and women were included in all studies. The mean age of patients with T2D was 58.7 years (range 54 to 66 years). The mean treatment period (follow-up period) was 38 weeks, and the mean duration of T2D was 10.8 (range 6.8 to 16) years. The mean HbA1c level and body mass index (BMI) at baseline were 8.5% (range 7.1% to 9.5%) and 30.2 (range 24.6 to 36.6), respectively. Moreover, the qualitative synthesis revealed a diverse range of pretreatment backgrounds among patients initiating basal insulins in included studies, with 27 RCTs specifically comparing efficacy and/or safety in insulin-naive patients (solely using oral anti-diabetic therapy), six assessing those already using basal insulin, three examining a population comprised of both insulin-naive individuals and those already using basal insulins, and eight RCTs focusing on patients utilizing a basal-bolus regimen before the commencement of the trial.

### Risk of bias assessment

All 44 randomized clinical trials were assessed for methodological quality, the results of which are presented in detail in **Supplementary Table 2**. Based on the assessment, two RCTs (4.5% of studies) were at high risk of bias, nine RCTs (19.5% of studies) posed some concerns at risk of bias, and 35 RCTs (76% of the studies) were placed in the low risk of bias category. More than two-thirds of the studies had a low risk of bias because of their standard design, conduct, analysis, and reporting. Most of the studies suffered from two domains: deviations from intended interventions and the measurement of the outcome. Deviation from the intervention was almost balanced among the groups in most of the studies, and the outcome assessors were aware of the type of insulin received by the patients in some studies. Sensitivity analysis was also conducted by excluding studies with a high risk of bias to examine the robustness of the model findings.

### Network plots

**Figure 2:**
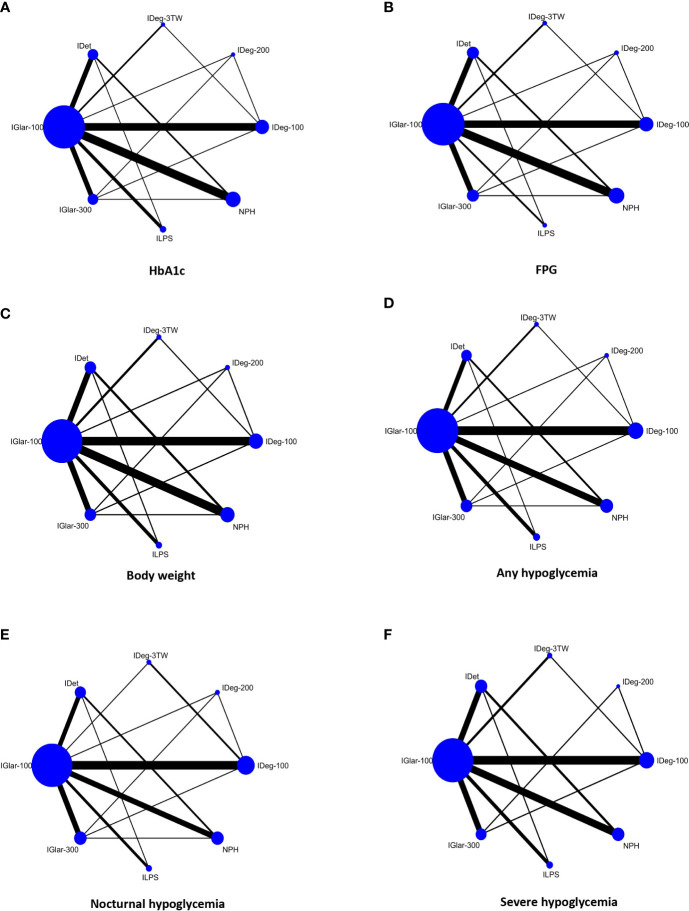
Network plots comparing basal insulins for change in HbA1c level **(A)**, FPG **(B)**, body weight **(C)**, overall/any hypoglycemia **(D)**, nocturnal hypoglycemia **(E)**, and severe hypoglycemia **(F)** in people with T2DM. Each node (circle) represents a basal insulin regimen, and its size is proportional to the number of participants randomly assigned to each treatment. The width of the line joining two nodes is proportional to the number of trials that directly compare the two respective treatments. Glargine, 100 U/mL (IGlar-100); Glargine, 300 U/mL (IGlar-300); Detemir (IDet); Degludec, 100 U/mL (IDeg-100); Degludec, 200 U/mL (IDeg-200); Thrice-weekly degludec (IDeg-3TW).

Network plots compare basal insulins regarding the effect on HbA1c level, FPG, body weight, any/overall, nocturnal, and severe hypoglycemia outcomes (six networks) (**Figure 2**). Basal insulins in the main analyses included IGlar-100, IGlar-300, IDet, IDeg-100, IDeg-200, IDeg-3TW, NPH, and ILPS. Among all comparisons, IGlar-100 was the most commonly used treatment (more participants were assigned in the trials). The number of RCTs comparing IGlar-100 against NPH and IDeg-100 was more than the other comparators in all networks.

### Evaluation of transitivity, heterogeneity, and inconsistency in all networks

The distribution of potential effect modifiers, including baseline HbA1c level and body weight as well as age and duration of T2D, were examined qualitatively; they were relatively the same across included studies in all six networks, so the transitivity assumption was accepted. Heterogeneity was assessed qualitatively by examining participants’ clinical and methodological characteristics across trials. Heterogeneity was also examined visually through the predictive interval plot. This plot assessed the uncertainty in the estimated treatment effects, which also includes the extent of heterogeneity (tau2 for HbA1c=0.01, for FPG=0.17, for body weight=0.23, for overall hypoglycaemia=0.02, for nocturnal hypoglycaemia=0.01, and for severe hypoglycaemia=0.001 indicating low heterogeneity among the studies in all networks).

Local consistency was evaluated using a loop-specific approach and confirmed (except for 2 of the 8 closed loops in HbA1c, 3 of the 8 closed loops for FPG, 2 of the 8 closed loops for body weight, 2 of the 8 closed loops for overall hypoglycemia, and 3 of the 8 closed loops for nocturnal hypoglycemia). In each network, most of the studies with small sample sizes and larger estimated standard errors caused local inconsistency. After excluding these trials from the networks (seven studies in the FPG network, four studies in body weight, three studies in overall hypoglycemia, and four studies in nocturnal hypoglycemia network), local consistency was established in each closed loop and led to more global consistency in some networks. Local consistency was also confirmed by comparing direct and indirect estimates through the node-splitting method (none of the *p*-values were statistically significant). Global consistency through the design-by-treatment interaction model showed no inconsistency in the networks (*p*-values for consistency ranged from 0.43 to 0.98 in networks of outcomes). The qualitative and quantitative assessments indicated that the network meta-analyses’ consistency was logical.

### Network meta-analyses estimates

#### Change in HbA1c level

The league table shows the random-effects network meta-analysis estimates (relative treatment effects, pooled mean difference) for comparison of basal insulins to change HbA1c level (**Table 1**). The findings of 44 RCTs (28 direct and indirect comparisons) indicated that IDeg-200 (pooled MD: -0.21% with 95% CI: -0.12% to -0.30%), IDeg-100 (Pooled MD:-0.15% with 95% CI: -0.02% to -0.27%), IDeg-3TW (Pooled MD: -0.27% with 95% CI: -0.08% to -0.45%), IDet (Pooled MD: -0.23% with 95% CI: -0.10% to -0.36%), and NPH (Pooled MD: -0.16% with 95% CI: -0.05% to -0.27%) are statistically significant effective than IGlar-100 in reducing HbA1c level. No other significant differences were identified in comparing basal insulins regarding changes in HbA1c levels. It seems that the observed difference between these insulins is not clinically significant, and therefore it can be said that almost all of them are equally effective in reducing the HbA1c level and do not have any particular preference over each other. Consistent results were observed when the included studies were restricted to RCTs with sample sizes of more than 100 patients in each study arm (excluding nine trials), parallel trials (excluding three trials), and RCTs with low risk of bias in separate sensitivity analyses. Visual inspection of the funnel plot did not reveal evidence of small-study effects (likely no publication bias).

**Table 1:** Random-effects network meta-analyses estimate for all direct and indirect evidence comparing basal insulins regarding the defined outcomes (A-F) for T2DM.

(A) Change in HbA1c level from baseline (MD, mean difference and 95% CI)							
**IDeg-200**							
-0.06 (-0.15,0.02)	**IDeg-100**						
0.06 (-0.11,0.22)	0.12 (-0.05,0.29)	**IDeg-3TW**					
-0.15 (-0.35,0.05)	-0.09 (-0.30,0.12)	-0.21 (-0.46,0.05)	**IGlar-300**				
**-0.21 (-0.30,-0.12)**	**-0.15 (-0.27,-0.02)**	**-0.27 (-0.45,-0.08)**	-0.06 (-0.28,0.16)	**IGlar-100**			
0.02 (-0.07,0.11)	0.08 (-0.03,0.19)	-0.04 (-0.20,0.12)	0.17 (-0.05,0.39)	**0.23 (0.10,0.36)**	**IDet**		
-0.10 (-0.23,0.03)	-0.04 (-0.20,0.12)	-0.16 (-0.37,0.05)	0.05 (-0.19,0.29)	0.11 (-0.04,0.26)	-0.12 (-0.28,0.04)	**ILPS**	
-0.05 (-0.13,0.03)	0.01 (-0.11,0.13)	-0.11 (-0.29,0.07)	0.10 (-0.12,0.32)	**0.16 (0.05,0.27)**	-0.07 (-0.19,0.05)	0.05 (-0.10,0.21)	**NPH**
(B) Change in FPG level from baseline (MD, mean difference and 95% CI)							
**IDeg-200**							
**5.27 (2.18,8.36)**	**IDeg-100**						
**6.65 (1.68,11.61)**	1.38 (-3.75,6.50)	**IDeg-3TW**					
-7.17 (-14.71,0.37)	**-12.44 (-20.34,-4.55)**	**-13.82 (-22.77,-4.87)**	**IGlar-300**				
**-4.16 (-7.95,-0.38)**	**-9.43 (-14.32,-4.55)**	**-10.81 (-17.05,-4.57)**	3.01 (-5.44,11.45)	**IGlar-100**			
-2.02 (-5.07,1.03)	**-7.29 (-11.17,-3.42)**	**-8.67 (-13.68,-3.65)**	5.15 (-2.94,13.24)	2.14 (-2.71,7.00)	**IDet**		
-3.37 (-8.35,1.60)	**-8.64 (-14.50,-2.79)**	**-10.02 (-17.04,-3.00)**	3.80 (-5.25,12.84)	0.79 (-4.72,6.30)	-1.35 (-7.18,4.48)	**ILPS**	
**-3.33 (-6.18,-0.49)**	**-8.60 (-12.79,-4.42)**	**-9.98 (-15.68,-4.28)**	3.84 (-4.24,11.92)	0.83 (-3.54,5.20)	-1.31 (-5.44,2.82)	0.04 (-5.60,5.68)	**NPH**
(C) Change in body weight from baseline (MD, mean difference and 95% CI)							
**IDeg-200**							
0.69 (-0.25,1.63)	**IDeg-100**						
-0.47 (-2.15,1.21)	-1.16 (-2.94,0.62)	**IDeg-3TW**					
**3.65 (0.85,6.45)**	**2.96 (0.06,5.86)**	**4.12 (0.87,7.36)**	**IGlar-300**				
1.12 (-0.02,2.26)	0.43 (-1.05,1.91)	1.59 (-0.44,3.62)	-2.53 (-5.55,0.49)	**IGlar-100**			
0.54 (-0.50,1.58)	-0.15 (-1.38,1.08)	1.01 (-0.53,2.56)	**-3.11 (-6.08,-0.14)**	-0.58 (-2.12,0.97)	**IDet**		
0.89 (-1.21,2.99)	0.20 (-2.11,2.50)	1.36 (-1.33,4.04)	-2.76 (-6.26,0.73)	-0.23 (-2.42,1.96)	0.34 (-2.00,2.69)	**ILPS**	
-0.01 (-0.95,0.92)	-0.71 (-2.03,0.62)	0.45 (-1.46,2.37)	**-3.66 (-6.61,-0.72)**	-1.13 (-2.42,0.15)	-0.56 (-1.95,0.83)	-0.90 (-3.16,1.36)	**NPH**
(D) Occurrence of overall/any hypoglycemia (OR, odds ratio and 95% CI)							
**IDeg-200**							
1.14 (0.84,1.55)	**IDeg-100**						
0.75 (0.46,1.22)	0.66 (0.38,1.13)	**IDeg-3TW**					
0.91 (0.71,1.18)	0.80 (0.56,1.13)	1.21 (0.74,1.99)	**IGlar-300**				
**0.78 (0.66,0.93)**	**0.69 (0.51,0.92)**	1.04 (0.66,1.65)	0.86 (0.71,1.04)	**IGlar-100**			
1.08 (0.87,1.35)	0.95 (0.71,1.27)	1.44 (0.88,2.35)	1.19 (0.91,1.54)	**1.38 (1.15,1.65)**	**IDet**		
0.86 (0.62,1.19)	0.75 (0.51,1.13)	1.14 (0.67,1.95)	0.94 (0.69,1.28)	1.10 (0.83,1.44)	0.80 (0.57,1.10)	**ILPS**	
**0.59 (0.46,0.75)**	**0.52 (0.37,0.73)**	0.78 (0.48,1.28)	**0.65 (0.51,0.81)**	**0.75 (0.63,0.90)**	**0.55 (0.42,0.70)**	**0.69 (0.50,0.94)**	**NPH**
(E) Occurrence of nocturnal hypoglycemia (OR, odds ratio and 95% CI)							
**IDeg-200**							
1.29 (0.93,1.78)	**IDeg-100**						
0.54 (0.28,1.05)	**0.42 (0.20,0.88)**	**IDeg-3TW**					
**0.72 (0.56,0.93)**	**0.56 (0.39,0.80)**	1.32 (0.65,2.67)	**IGlar-300**				
**0.71 (0.59,0.84)**	**0.55 (0.40,0.75)**	1.30 (0.66,2.57)	0.99 (0.82,1.19)	**IGlar-100**			
0.93 (0.75,1.16)	**0.72 (0.54,0.98)**	1.72 (0.86,3.43)	**1.30 (1.01,1.67)**	**1.32 (1.11,1.56)**	**IDet**		
**0.51 (0.36,0.72)**	**0.40 (0.26,0.61)**	0.94 (0.45,1.98)	**0.71 (0.52,0.98)**	**0.72 (0.53,0.98)**	**0.55 (0.39,0.77)**	**ILPS**	
**0.41 (0.32,0.53)**	**0.32 (0.22,0.45)**	0.75 (0.37,1.52)	**0.57 (0.45,0.71)**	**0.58 (0.48,0.69)**	**0.44 (0.34,0.56)**	0.80 (0.57,1.13)	**NPH**
(F) Occurrence of severe hypoglycemia (OR, odds ratio and 95% CI)							
**IDeg-200**							
1.82 (0.82,4.02)	**IDeg-100**						
0.67 (0.12,3.80)	0.37 (0.06,2.32)	**IDeg-3TW**					
0.86 (0.46,1.63)	0.47 (0.21,1.05)	1.28 (0.22,7.51)	**IGlar-300**				
0.69 (0.44,1.10)	**0.38 (0.20,0.75)**	1.03 (0.19,5.73)	0.81 (0.52,1.25)	**IGlar-100**			
0.74 (0.41,1.34)	**0.41 (0.23,0.70)**	1.10 (0.19,6.37)	0.86 (0.47,1.55)	1.06 (0.71,1.59)	**IDet**		
**0.27 (0.09,0.86)**	**0.15 (0.04,0.52)**	0.40 (0.05,3.02)	**0.32 (0.11,0.92)**	0.39 (0.14,1.12)	0.37 (0.12,1.14)	**ILPS**	
**0.51 (0.29,0.90)**	**0.28 (0.13,0.59)**	0.76 (0.13,4.34)	0.59 (0.35,1.00)	0.74 (0.53,1.02)	0.69 (0.41,1.16)	1.87 (0.63,5.61)	**NPH**
High Certainty Based on GRADE Ratings							
Moderate Certainty Based on GRADE Ratings							
Low Certainty Based on GRADE Ratings							
Very Low-Certainty Based on GRADE Ratings							

Diagonal cells contain the names of competing treatments. The tables show column-to-row mean differences or odds ratio with 95% CIs for change in HbA1c level from baseline, change in FPG from baseline, change in body weight from baseline, and odds of overall, nocturnal, and severe hypoglycemia. Statistically significant differences are bolded and favor the column-defining treatment (for example i.e., the treatment in the column is associated with a less weight gain than the treatment in the row).

#### Change in FPG level

Forty RCTs that compared eight basal insulins were included in the network meta-analysis. Based on an extended forest plot for direct evidence and a predictive interval plot using direct and indirect evidence, except for a few comparisons, there is no clinically significant difference between basal insulins regarding the effect on FPG level in T2D.

Estimates from network meta-analysis for all 28 direct and indirect comparisons (league Table, **Table 1**) showed that IDeg-3TW and IDeg-100 compared to IGlar-300 (Pooled MD:-13.8 mg/dL with 95% CI: -22.7 to -4.8 and -12.4 mg/dL with 95% CI: -20.3 to -4.5 respectively), IGlar-100 (Pooled MD: -10.8 mg/dL with 95% CI: -17.05 to -4.5 and -9.9 mg/dL with 95% CI: -14.3 to -4.5 respectively), IDet (Pooled MD: -8.6 with 95% CI: -13.6 to -3.6 and -7.3 mg/dL with 95% CI: -11.2 to -3.4 respectively), ILPS (Pooled MD: -10.1 with 95% CI: -17.1 to -3.0 and -8.6 mg/dL with 95% CI: -14.5 to -2.8 respectively), and NPH (Pooled MD: -9.9 with 95% CI: -15.6 to -4.3 and -8.6 mg/dL with 95% CI: -12.8 to -4.4 respectively) were slightly more effective in reducing FPG level, but this magnitude of difference was not clinically important. Results were consistent in sensitivity analyses when included studies were restricted to RCTs with a low risk of bias and a sample size of more than 100 patients in each treatment arm. Inspection of the funnel plot indicated no evidence of publication bias, implying no evidence of small-study effects in the network.

#### Change in body weight

Data on the changes in body weight from baseline were available from 36 RCTs. The league table findings from the network meta-analysis for all 28 direct and indirect comparisons of eight basal insulins indicated that patients with T2D treated with IGlar-300 were significantly associated with less body weight gain compared to IDeg- 3TW (pooled MD: -4.12 kg with 95% CI: -0.87 kg to -7.36 kg), IDeg-100 (pooled MD: -2.96 kg with 95% CI: -0.06 kg to -5.8 kg), IDeg- 200 (Pooled MD: -3.65 kg with 95% CI: -0.85 kg to -6.45 kg), NPH (Pooled MD: -3.66 kg with 95% CI: -0.72 kg to -6.61 kg), and IDet (Pooled MD: -3.1 kg with 95% CI: -0.14 kg to -6.08 kg) (**Table 1**). The weight gain by IGlar-300 compared to other basal insulins is considered important from a clinical point of view. It might represent this insulin as the preferred choice regarding the less harmful effect on body weight (where the weight gain is of lower magnitude). Sensitivity analyses demonstrated the consistency of the results, indicating that the network meta-analysis model was robust. The comparison-adjusted funnel plot showed publication bias was unlikely.

### Overall (any) hypoglycemia

Forty-three randomized clinical trials were considered in the network meta-analysis to compare the effect of eight basal insulins on hypoglycemia. Overall (any) hypoglycemia was defined as typical symptoms associated with hypoglycemia with or without a plasma glucose level (3.9 mmol/L or less) or a confirmed plasma glucose level of 3.9 mmol/L or less. In general, findings from the extended forest plot for direct evidence and the predictive interval plot using direct and indirect evidence showed a significant difference in the incidence of overall/any hypoglycemia between some comparisons.

The findings from the network meta-analysis of 43 RCTs (league table) indicated that the use of all basal insulins except IDeg-3TW was associated with significantly lower odds of hypoglycemia compared to NPH (ranging from 25% (IGlar-100) to 48% (IDeg-100)). The odds of any hypoglycemia in patients with T2D treated with IDeg-200 (pooled odds ratio: 0.78 with 95% CI: 0.66 to 0.93) and IDeg-100 (pooled OR: 0.69 with 95% CI: 0.51 to 0.92) were significantly lower than IGlar-100. IDet was also associated with lower odds of overall hypoglycemia compared to IGlar-100. No significant differences were observed for other treatment comparisons (**Table 1**). Both findings of relative ranking and network meta-analysis estimates showed that IDeg-100, IDeg-200, and IDet could be preferred choices, and IDeg-3TW and NPH should be the least favored options for attaining less hypoglycemia. Small-study effects were unlikely in assessing comparison-adjusted funnel plots in the network.

### Nocturnal hypoglycemia

To compare basal insulins to investigate their effect on nocturnal hypoglycemia, 40 RCTs contributed to the network. All trials included in the network meta-analysis defined nocturnal hypoglycemia as any hypoglycemic event between bedtime and waking (usually between 12:00 PM and 6:00 AM). Extended forest and predictive interval plots showed significant differences in the occurrence of nocturnal hypoglycemia in some comparisons among basal insulins.

Administration of all basal insulins except IDeg-3TW compared to NPH and ILPS significantly reduced the odds of nocturnal hypoglycemia (**Table 1**). IGlar-100 and IGlar-300 significantly increased the odds of nocturnal hypoglycemia compared to IDet (pooled OR: 1.32 with 95% CI: 1.11 to 1.56; and pooled OR: 1.30 with 95% CI: 1.01 to 1.67 respectively). Patients with T2D treated with IDeg-100 had significantly lower odds of nocturnal hypoglycemia compared to other basal insulins [except IDeg-200) (ranging from 28% (against IDet) to 68% (against NPH)]. As with overall hypoglycemia, it can be stated that IDeg-100, IDeg-200, and IDet could be the first choices for obtaining less nocturnal hypoglycemia, and the use of NPH and IDeg-3TW should be the last priority in terms of prevention of nocturnal hypoglycemia. Results were consistent in sensitivity analyses when only low-risk-of-bias trials, parallel designs, and studies with more than 100 patients per arm were included. There was no evidence of publication bias.

### Severe hypoglycemia

Thirty-five RCTs were included in the network meta-analysis to assess basal insulins’ effect on severe hypoglycemic events. All studies consistently defined severe hypoglycemia as an event that required third-person assistance. Considerable difference in the occurrence of severe hypoglycemia was observed in some comparisons among basal insulins based on extended forest plots using direct evidence and predictive interval plots using direct and indirect evidence.

Treatment with IDeg-100, IDeg-200, and IGlar-300 was associated with significantly reduced odds of severe hypoglycemia compared to NPH and ILPS. IDeg-100 significantly resulted in lower odds of severe hypoglycemia compared to IGlar-100, IDet, ILPS, and NPH (pooled OR: 0.38 with 95% CI: 0.20 to 0.75; pooled OR: 0.41 with 95% CI: 0.23 to 0.70; pooled OR: 0.15 with 95% CI: 0.04 to 0.52; and pooled OR: 0.28 with 95% CI: 0.13 to 0.59) respectively (**Table 1**). Both relative ranking and league table findings indicated that the first choices that result in less severe hypoglycemia could be IDeg-100, IDeg-200, and IGlar-300, and the last options are NPH, ILPS, and IDeg-3TW. The network meta-analysis model was robust, and findings were consistent in sensitivity analyses restricted to large trials and studies with low risk of bias. Small-study effects were unlikely in assessing comparison-adjusted funnel plots in the network.

To achieve accurate and reliable findings, network meta-regressions were performed considering HbA1c, body mass index, and age of patients at baseline. However, due to the homogeneity among studies and consistency of the networks, no significant differences were observed in the estimated treatment effects (data not shown).

### Summary of findings and certainty of the evidence

Despite the statistically significant difference in the reduction of HbA1c among specific network comparisons, these differences did not reach the minimal clinically important differences (more than 0.5%). Although the evidence is relatively uncertain for this outcome (most comparisons had low to very low certainty), the current effect estimates differ by such a small magnitude that future studies are unlikely to change this conclusion. Similarly, the effects of different basal insulins on the reduction of FPG were comparable, with statistically significant differences ranging between 3 to 13 mg/dl.

Regarding the effect of insulins on patients’ weight, IGlar-300 and IGlar-100 may be associated with less weight gain than other basal insulins. However, these differences were slight (3-4 kg), and the effect estimates were imprecise (low to very low-quality evidence).

Moderate to high certainty evidence showed that IDeg-100 and IDeg-200 were the safest options when considering hypoglycemia-related outcomes, followed by IDet for nocturnal and overall hypoglycemia. The next safe option regarding nocturnal and overall hypoglycemia was IDeg-3TW (low-certainty evidence). Based on moderate-quality evidence, ILPS, IGlar-100, and IGlar-300 have similar profiles concerning overall hypoglycemia. However, ILPS was associated with higher rates of nocturnal hypoglycemia than IGlar-100 (moderate-certainty evidence). The intermediate-acting insulin NPH was associated with the highest odds of overall and nocturnal hypoglycemia (moderate-certainty evidence). **Table 2** presents the summary of findings using the GRADE minimally-contextualized framework.

**Table 2:** The summary of findings table with the certainty of the rankings using the GRADE minimally-contextualized framework.

Change in HbA1c	Change in FPG	Change in Body Weight	Severe Hypoglycemia	Overall Hypoglycemia	Nocturnal Hypoglycemia
IDeg-200	IDeg-3TW	IGlar-300	IDeg-100	IDeg-100	IDeg-100
IDeg-100	IDeg-100	IGlar-100	IDeg-200	IDeg-200	IDeg-200
IDeg-3TW	IDeg-200	ILPS	IGlar-300	IDet	IDet
IDet	IGlar-100	IDet	IDet	IDeg-3TW	IDeg-3TW
NPH	IGlar-300	IDeg-100	IGlar-100	IGlar-300	IGlar-300
IGlar-300	IDet	IDeg-200	IDeg-3TW	IGlar-100	IGlar-100
IGlar-100	ILPS	NPH	NPH	ILPS	ILPS
ILPS	NPH	IDeg-3TW	ILPS	NPH	NPH
**High to Moderate Certainty Evidence**			**Low to Very Low Certainty Evidence**		
Among the most effective/least harmful			Possibly among the most effective		
Not convincingly different from standard treatment (IGlar-100)			Possibly not convincingly different from standard treatment (IGlar-100)		
Among the most harmful/least effective			Possibly among the most harmful/least effective		

## Discussion

The current study’s findings enable patients and healthcare practitioners to make informed choices to individualize insulin therapy, depending on whether weight gain or hypoglycemia is the most significant concern in the shared decision-making process. The control and management of T2D are always complex and require careful consideration of patient-related factors such as capability and desire for self-care, co-morbidities, and costs (80). Type 2 diabetes is a progressive disease, and many patients require injectable medications, including insulin, at some point in the course of the disease (81, 82). As a result, decision-makers and clinicians must rely mainly on research evidence and clinical judgment in choosing a specific basal insulin among all available basal insulins. By conducting a network meta-analysis of the current evidence, we try to address these issues and provide evidence for decision-makers and clinicians to select the most appropriate option for their patients. For instance, older, frail patients who are at the highest risk of hypoglycemia may choose to prioritize insulins that have a lower risk of hypoglycemia. Conversely, insulins that have a lower propensity to cause weight gain may be the preferred option in patients who are concerned about the complications of becoming overweight or obese.

We combined direct and indirect evidence to compare the effects of basal insulin regimens on HbA1c reduction, weight gain, and hypoglycemic events. Regarding glycemic control, subtle yet statistically significant differences were observed when comparing the efficacy of distinct basal insulin regimens in reducing HbA1C. It is crucial to emphasize that relying solely on the interpretations derived from a network meta-analysis may not align perfectly with clinical practice objectives. Various guidelines, including the recent National Institute for Health and Care Excellence (NICE) recommendations for diabetes management, have established a more than 0.5% change in HbA1C as the minimal clinically significant threshold (83). Our analysis demonstrated that neither the crude calculated differences nor their associated confidence intervals surpassed this threshold. Consequently, despite statistical significance, we deemed these observed differences clinically insignificant.

Regarding changes in body weight, IGlar-300 was associated with the least weight gain compared to insulin degludec, detemir, and NPH. Treatment with NPH was associated with the highest odds of overall and nocturnal hypoglycemia compared to all other basal insulins, while patients treated with IDeg-100, IDeg-200, IDet, and IGlar-300 were less likely to experience overall and nocturnal hypoglycemia than the patients treated with other basal insulin regimens. Treatment regimens that consisted of NPH and ILPS were more likely to cause severe hypoglycemia, whereas treatment with IDeg-100 was associated with a significant reduction in the odds of severe hypoglycemia compared to IGlar-100 and IDet, NPH, and ILPS. NPH insulin exhibits lower efficacy in weight management and has a higher propensity to induce hypoglycemic events compared to other basal insulins. These observations are consistent with each other since the increased weight gain associated with NPH may be partially attributed to the overconsumption of food triggered by the hypoglycemic episodes elicited by this drug.

Choosing the optimal treatment among intermediate, long, and ultra-long-acting basal insulins in people with T2D should be based on the effects of insulins on clinically relevant parameters, including unmet needs (84). The present study’s findings indicate that newer-generation basal insulins do not seem to improve glycemic control compared to first-generation basal insulins such as insulin glargine-100, insulin detemir, or even NPH insulin. However, where less weight gain is a priority, insulin glargine-300 is the superior choice. Also, considering nocturnal hypoglycemia, administration of insulin degludec-100, insulin degludec-200, or insulin glargine-300 is associated with the lowest incidence odds.

We did not find a significant difference between IDeg-100 and IDeg-200 in the present study. Therefore, both could be considered potential clinical practice options when indicated (24). Challenges remain regarding the clinical use of ILPS, as it is associated with an increased risk of severe and nocturnal hypoglycemia without relative beneficial effects on blood glucose control or body weight. It is also recommended to consider patients’ compliance with treatment and cardiovascular safety in deciding on the best treatment options (85–87).

To our knowledge, cardiovascular outcomes have not been compared in patients receiving different basal insulins. However, a recent meta-analysis by Rados et al. demonstrated that basal insulins, regardless of their type, are not associated with an increased risk of either cardiovascular events incidence or all-time cardiovascular mortality compared to other potential T2D treatments (88). Further investigation and comparison of different basal insulin effects on cardiovascular outcomes can aid physicians in devising personalized treatment plans best suited to individual patients’ unique needs.

Patients’ adherence significantly affects the efficacy of glucose-lowering agents. Although treatments demonstrate comparable effectiveness in randomized controlled trials, it’s crucial to recognize that trial participants are a selectively motivated and highly adherent group. As a result, findings from clinical trials may not consistently represent the broader characteristics of the general population (89, 90).

Experience from real-world data suggests that T2D treatment adherence is greatly influenced by the ease of drug administration, dosing frequency, treatment complexity, out-of-pocket costs, and incident hypoglycemia (91). For example, non-adherence is more significant with injectable drugs than with oral medications, as drug injections are more challenging and can be inconvenient for patients (92). Similarly, adherence is better for insulin pens than for insulin administered by vial and syringe (91, 93). Hence, when comparing ease of use between medication groups, differences between insulin administered by vial and syringe versus insulin done through the pen should be considered.

As T2D advances, individuals on NPH insulin may require more than one injection per day (5), a factor that could compromise treatment adherence. Considering its suboptimal performance in various aspects of efficacy and safety, the utilization of NPH insulin is recommended primarily in situations where cost considerations weigh significantly on the patient’s decision-making process.

In a similar design to the current study, Madenidou et al. conducted a network meta-analysis on RCTS assessing the efficacy and safety of basal insulins in T2D patients in 2018. They indicated that the difference in efficacy among basal insulin analogs is minimal (94). The present study’s findings suggest the same regarding the efficacy of basal insulins. They also observed that IDet was associated with less weight gain than other basal insulins, and IGlar-300 had a more favorable weight gain profile than IDeg-100, IDeg-200, Ideg-3TW, and IGlar-100. Similarly, we report less weight gain associated with IGlar 300 than IDeg-100, IDeg-200, and Ideg-3TW. However, our network meta-analysis showed the superiority of IGlar 300 to IDet regarding weight gain. Moreover, Madenidou et al. indicated that the incidence of severe hypoglycemia did not differ between basal insulins, except for ILPS, which was associated with a higher risk of hypoglycemia compared to other basal insulin regimens. It should be noted that the researchers included Glargine biosimilars and did not include NPH insulin in their review. In contrast, the present network meta-analysis included NPH insulin as well as larger randomized clinical trials reported in recent years that are expected to further contribute to the external validity or generalizability of the findings in the current study. Insulin glargine and glargine biosimilars were similar in terms of efficacy and safety related to the reduction of HbA1c level, effect on body weight, or occurrence of hypoglycemia in various reported studies as well as in a comprehensive systematic review (94, 95); hence, glargine biosimilars were not included in our review and analyses. In 2016, Freemantle et al. conducted a network meta-analysis on randomized clinical trials comparing different basal insulins (including IGlar-300, IGlar-100, NPH, Detemir, and Degludec) and premixed insulins with each other in T2D patients (96). They reported the superiority of IGlar-300 over NPH and premixed insulins in reducing hypoglycemic events. They also stated that the safety of other basal insulins is comparable. In line with their findings, we also report the superiority of IGlar-300 over NPH. However, the results of the current meta-analysis suggest the superiority of IDeg-100 and IDeg-200 in reducing the odds of nocturnal hypoglycemia. This might be due to the fact that the findings of more recent large sample RCTs comparing Degludec and Glargine insulins have been released since they conducted their network meta-analysis. It is also worth mentioning that Freemantle et al. included premixed insulins in their network. In contrast, our analysis focused exclusively on basal insulins, contributing to a more targeted investigation of this specific insulin category.

### Limitations and strengths

There are some limitations in the present study. The findings of the network meta-analysis should be interpreted with caution in the context of limitations of the available data. The comparative effectiveness of newer basal insulins has been based mainly on indirect comparisons. Therefore, any inferences about the favorable effect of second-generation basal insulins should be interpreted with caution. Differences in the use of background glucose-lowering medications among some trials are important to consider. Although we tried to maintain homogeneity among the participants, the efforts did not eliminate heterogeneity ideally. Measurement of specific outcomes between intervention groups has been similar in the reviewed studies. Still, in some cases, the outcome assessors were aware of the type of insulin received by the patients. Therefore, the outcome measurement may be affected by the knowledge of the type of insulin received. Also, the variation in daily insulin dose plans among the trials included in the analyses should be considered. Also, the present network meta-analysis has the same limitations and common challenges present in other network meta-analyses (97), indicating that the findings should be interpreted cautiously. We took steps to minimize these limitations by applying methods proposed by GRADE (32–37) and presented the findings’ certainty and the effect estimates to avoid misinterpretation of the results.

The present study also has some strengths. Publication bias was most unlikely in our systematic review because we have searched multiple sources, including abstracts and trial registries. The favorable quality of the included studies (76% of the trials had a low risk of bias) and the multinational and multicenter nature of the majority of trials with a considerable number of participants strengthen the validity and confidence of the research findings. The low heterogeneity of the participants (especially in terms of the main and important baseline variables), study designs, and the clinical characteristics of the patients led to the acceptable consistency of the networks and relatively realistic findings.

## Conclusions

Findings of the combination of direct and indirect evidence with acceptable quality indicate that basal insulin regimens are comparable in glycemic control in people with T2D. Insulin glargine, 300 U/mL, may be associated with a slightly less severe weight gain than other basal insulins. Insulin degludec 100 U/mL, degludec 200 U/mL, detemir, and glargine 300 U/mL are preferred options when hypoglycemia is the primary concern.

## Data availability statement

The original contributions presented in the study are included in the article/**Supplementary Material**, further inquiries can be directed to the corresponding author/s.

## Author contributions

MD: Writing – original draft, Visualization, Software, Methodology, Investigation, Data curation, Writing – review & editing, Conceptualization. MS: Writing – original draft, Writing – review & editing, Software, Methodology, Investigation, Formal analysis, Data curation. FB: Writing – review & editing, Visualization, Investigation, Formal analysis. ZM: Writing – review & editing, Supervision, Methodology. LJ: Writing – review & editing, Supervision, Formal analysis. SM: Writing – review & editing, Supervision, Methodology. YL: Writing – review & editing, Methodology. FI: Writing – review & editing, Writing – original draft, Supervision, Methodology. HB: Writing – review & editing, Supervision, Conceptualization. MK: Writing – review & editing, Writing – original draft, Supervision, Methodology, Investigation, Conceptualization.
