# Supplementary material for: Efficacy and safety of basal insulins in people with type 2 diabetes mellitus: a systematic review and network meta-analysis of randomized clinical trials
Source: Front Endocrinol (Lausanne). 2024 Mar 21;15:1286827. doi: 10.3389/fendo.2024.1286827 (PMC10997219; doi:10.3389/fendo.2024.1286827)
Supplement: Supplementary file 1 [file Table_1.docx]

**Supplementary table1.** Main characteristics of the 46 randomized controlled trials comparing basal insulin regimens for treatment of type 2 diabetes.

| **Study, year** | **Country** | **Duration of DM (years)** | **Follow up (weeks)** | **Baseline BMI**  **(kg/m²)** | **Baseline HbA1c (%)** | **Mean Age (years)** | **ROB** | **Treatment arm (1)** | **Sample size** | **Treatment arm (2)** | **Sample size** |
| --- | --- | --- | --- | --- | --- | --- | --- | --- | --- | --- | --- |
| Goldenberg, 2021 | USA, Can, Pol, S Afr, and Slovak | 15 | 16 | 32.2 | 7.6 | 62.8 | Low risk | IDeg-100 | 224 | IGlar-100 | 224 |
| Ling, 2021 | China | 13 | 24 | 24.8 | 8.92 | 58.4 | Some concerns | IGlar-300 | 23 | NPH | 23 |
| Philis-Tsimikas, 2020 | 11 countries | 15 | 88 | 31.6 | 7.6 | 62.9 | Low risk | IDeg-200 | 805 | IGlar-300 | 804 |
| Ji, 2020 | China, Korea, and Taiwan | 10.6 | 26 | 25.2 | 8.6 | 58.3 | Some concerns | IGlar-300 | 397 | IGlar-100 | 201 |
| Rosenstock, 2018 | 16 countries | 10.6 | 24 | 31.5 | 8.6 | 60.5 | Low risk | IGlar-300 | 462 | IDeg-100 | 462 |
| Ritzel, 2018 | 18 countries | 15.3 | 26 | 31 | 8.2 | 71 | Low risk | IGlar-300 | 508 | IGlar-100 | 506 |
| Wysham, 2017 | USA | 14.1 | 32 | 32.2 | 7.6 | 61.4 | Low risk | IDeg-100 | 361 | IGlar-100 | 360 |
| Terauchi, 2017 | Japan | 14 | 48 | 25.3 | 8.02 | 60.8 | Some concerns | IGlar-300 | 121 | IGlar-100 | 120 |
| Bolli, 2017 | 15 countries (12 Europe) | 9.8 | 48 | 33 | 8.54 | 57.7 | Some concerns | IGlar-300 | 432 | IGlar-100 | 430 |
| Aso, 2017 | Japan | 11.5 | 24 | 24.6 | 8.86 | 64.3 | Some concerns | IDeg-100 | 31 | IGlar-100 | 12 |
| Pan, 2016 | USA, Can, Bra, Ukr, Chi, S Afr | 8 | 26 | 27.2 | 8.3 | 56.3 | Low risk | IDeg-100 | 555 | IGlar-100 | 278 |
| Yki-Jarvinen, 2015 | 13 countries (8 Europe) | 12.6 | 52 | 34.8 | 8.24 | 58.2 | Low risk | IGlar-300 | 403 | IGlar-100 | 405 |
| Riddle, 2015 | 13 countries (3 N Am, 9 Europe, S Afr) | 16 | 52 | 36.6 | 8.15 | 60 | Low risk | IGlar-300 | 404 | IGlar-100 | 400 |
| Home, 2015 | 16 countries (Europe, Asia, Mid East, S Am) | 9.2 | 36 | 29.8 | 8.2 | 57.3 | Low risk | IGlar-100 | 352 | NPH | 349 |
| Rosenstock, 2014 | USA and Canada | 10.7 | 260 | 34.3 | 8.4 | 55.1 | Low risk | IGlar-100 | 498 | NPH | 486 |
| Bode, 2014 | USA | 12.8 | 22 | 33.3 | 8.2 | 59.8 | Some concerns | IDeg-200 | 186 | IDeg-100 | 187 |
| Arakaki, 2014 | USA and Puerto Rico | 9.9 | 24 | 34.9 | 8.22 | 56.4 | Low risk | ILPS | 171 | IGlar-100 | 168 |
| Zinman, 2013 | 7 countries (Can, Cze, Isr, Slov, S Af, UK, USA) | 8.8 | 26 | 32.45 | 8.25 | 58.1 | Low risk | IDeg-3TW | 229 | IGlar-100 | 230 |
| Rodbard, 2013 | 12 countries (European, USA and Canada) | 9 | 104 | 31.25 | 8.2 | 59 | Low risk | IDeg-100 | 773 | IGlar-100 | 257 |
| Onishi, 2013 | 6 Asian countries (Hong, Jap, Kor, Malay, Taiw, Thail) | 11.6 | 26 | 25 | 8.5 | 58.6 | Some concerns | IDeg-100 | 289 | IGlar-100 | 146 |
| Meneghini, 2013 | 5 countries (USA, Arg, Ind, Kor, Thail) | 8.2 | 26 | 29 | 7.92 | 57.3 | Low risk | IDet | 226 | IGlar-100 | 227 |
| Meneghini, 2013 | 14 countries (Europe, Asia, Africa, Arg, Mexico) | 10.6 | 26 | 29.6 | 8.4 | 56.4 | Low risk | IDeg-100 | 228 | IGlar-100 | 230 |
| Gough, 2013 | 8 countries (Europe, S Africa, UK, UAS, Can) | 8.3 | 26 | 32.4 | 8.3 | 57.6 | Low risk | IDeg-200 | 228 | IGlar-100 | 229 |
| Garber, 2012 | 12 countries (9 Europe, USA, S Afr, Hong K) | 13.5 | 52 | 32.2 | 8.3 | 58.6 | Low risk | IDeg-100 | 744 | IGlar-100 | 248 |
| Zinman, 2011 | USA, Canada, India, and S Africa | 6.8 | 16 | 29.5 | 8.7 | 54.2 | Low risk | IDeg-3TW | 62 | IGlar-100 | 62 |
| Zinman, 2011 | USA, Canada, India, and S Africa | 6.8 | 16 | 29.5 | 8.7 | 54.2 | Low risk | IDeg-100 | 60 | IGlar-100 | 62 |
| Zinman, 2011 | USA, Canada, India, and S Africa | 6.8 | 16 | 29.5 | 8.7 | 54.9 | Low risk | IDeg-3TW | 62 | IDeg-100 | 60 |
| Koivisto, 2011 | 10 European countries | 11 | 24 | 33.2 | 8.8 | 59.8 | Low risk | ILPS | 179 | IGlar-100 | 180 |
| Fadini, 2011 | Italy | - | 12 | 28.3 | 8.7 | 66.1 | Some concerns | IDet | 21 | IGlar-100 | 21 |
| Swinnen, 2010 | 20 (14 Euro, Can, Bra, Aus, Ind, Kor, Taiw) | 9.9 | 24 | 30.1 | 8.7 | 58.4 | Low risk | IDet | 486 | IGlar-100 | 478 |
| Strojek, 2010 | 10 countries (Asia, Africa, Europe, USA) | 9.8 | 24 | 31.15 | 8.67 | 57.7 | Low risk | ILPS | 102 | IGlar-100 | 229 |
| Fogelfeld, 2010 | 9 (3 Euro, Arg, Ind, Kor, Mex, Taiw, USA) | 9.3 | 24 | 30.1 | 8.8 | 56 | Low risk | ILPS | 209 | IDet | 202 |
| Raskin, 2009 | USA | 12.3 | 26 | 32.7 | 8.4 | 55.8 | High risk | IDet | 254 | IGlar-100 | 131 |
| Rosenstock, 2008 | Denmark, Austria, UK, USA | 9.1 | 52 | 30.6 | 8.6 | 58.9 | Low risk | IDet | 268 | IGlar-100 | 275 |
| Hollander, 2008 | European Union and USA | 13.5 | 52 | 31.6 | 8.7 | 58.5 | Low risk | IDet | 214 | IGlar-100 | 105 |
| Pan CY, 2007 | 10 East Asian (Chi, Indo, Kor, Malay, Pak..) | 10.2 | 24 | 25 | 9.04 | 56.1 | Low risk | IGlar-100 | 220 | NPH | 223 |
| Yokoyama, 2006 | Japan | 13 | 24 | 26.3 | 7.1 | 61.5 | Some concerns | IGlar-100 | 31 | NPH | 31 |
| Yki-Jarvinen, 2006 | Finland and UK | 9 | 36 | 31.7 | 9.5 | 56.5 | Low risk | IGlar-100 | 61 | NPH | 49 |
| Hermansen, 2006 | 10 European countries | 9.7 | 24 | 29 | 8.5 | 60.9 | Low risk | IDet | 230 | NPH | 232 |
| Eliaschewitz, 2006 | 10 Latin American countries | 10.6 | 24 | 27.2 | 9.1 | 56.6 | Low risk | IGlar-100 | 231 | NPH | 250 |
| Haak, 2005 | 5 European countries (Ger, Ita, Den, Aust, UK) | 13.3 | 26 | 30.4 | 7.9 | 60.4 | Low risk | IDet | 315 | NPH | 155 |
| Riddle, 2003 | USA and Canada | 8.7 | 24 | 32.4 | 8.6 | 55.5 | Low risk | IGlar-100 | 367 | NPH | 389 |
| Massi Benedetti, 2003 | 14 European countries and South Africa | 10.4 | 52 | 29.1 | 9 | 59.5 | High risk | IGlar-100 | 289 | NPH | 281 |
| Fritsche, 2003 | 13 European countries | 8.7 | 24 | 28.8 | 9.1 | 61 | Low risk | IGlar-100 | 227 | NPH | 232 |
| Yki-Jarvinen, 2000 | Finland and Germany | 10 | 52 | 28.9 | 9 | 59 | Low risk | IGlar-100 | 214 | NPH | 208 |
| Esposito, 2008 | Italy | 8 | 36 | 29.6 | 8.75 | 54.35 | Low risk | ILPS | 55 | IGlar-100 | 55 |

**Supplementary table2.** Revised Cochrane risk of bias assessment tool (ROB-2) for included RCTs

| **Study** | **Randomization process** | **Deviations from intended interventions** | **Missing outcome data** | **Measurement of the outcome** | **Selection of the reported results** | **Overall Bias** |  |
| --- | --- | --- | --- | --- | --- | --- | --- |
| Ling J, 2021 | 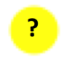 | 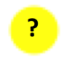 | 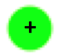 | 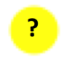 | 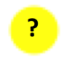 | 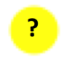 |  |
| Goldenberg RM, 2021 | 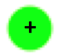 | 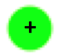 | 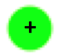 | 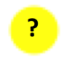 | 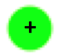 | 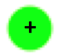 |  |
| Philis-Tsimikas A, 2020 | 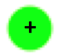 | 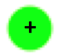 | 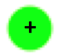 | 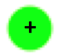 | 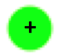 | 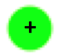 |  |
| Ji L, 2020 | 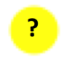 | 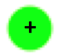 | 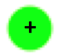 | 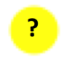 | 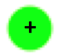 | 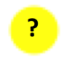 |  |
| Rosenstock J, 2018 | 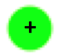 | 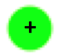 | 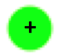 | 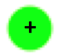 | 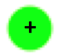 | 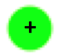 |  |
| Ritzel R, 2018 | 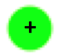 | 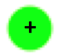 | 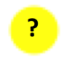 | 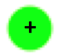 | 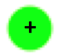 | 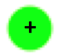 |  |
| Wysham C, 2017 | 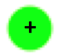 | 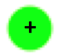 | 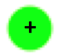 | 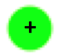 | 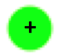 | 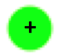 |  |
| Terauchi Y, 2017 | 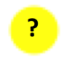 | 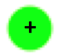 | 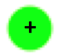 | 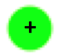 | 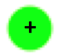 | 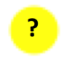 |  |
| Bolli GB, 2017 | 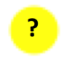 | 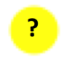 | 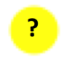 | 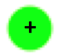 | 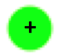 | 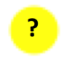 |  |
| Aso Y, 2017 | 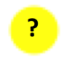 | 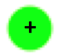 | 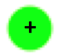 | 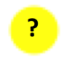 | 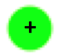 | 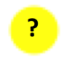 |  |
| Pan C, 2016 | 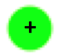 | 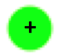 | 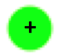 | 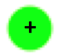 | 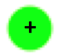 | 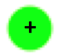 |  |
| Yki-Jarvinen H, 2015 | 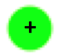 | 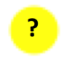 | 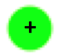 | 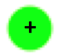 | 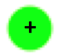 | 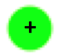 |  |
| Riddle MC, 2015 | 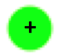 | 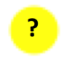 | 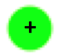 | 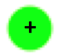 | 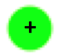 | 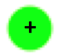 |  |
| Home PD, 2015 | 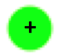 | 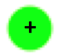 | 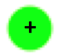 | 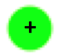 | 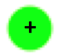 | 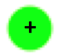 |  |
| Rosenstock J, 2014 | 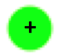 | 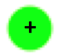 | 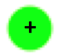 | 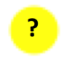 | 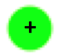 | 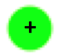 |  |
| Bode BW, 2014 | 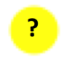 | 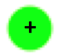 | 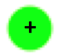 | 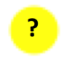 | 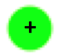 | 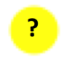 |  |
| Arakaki RF, 2014 | 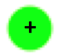 | 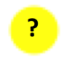 | 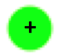 | 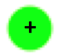 |  |  |  |
| Zinman B, 2013 |  |  |  |  |  |  |  |
| Rodbard HW, 2013 |  |  |  |  |  |  |  |
| Onishi Y, 2013 |  |  |  |  |  |  |  |
| Meneghini L, 2013 |  |  |  |  |  |  |  |
| Meneghini L, 2013 |  |  |  |  |  |  |  |
| Gough SC, 2013 |  |  |  |  |  |  |  |
| Garber AJ, 2012 |  |  |  |  |  |  |  |
| Zinman B, 2011 |  |  |  |  |  |  |  |
| Koivisto V, 2011 |  |  |  |  |  |  |  |
| Fadini GP, 2011 |  |  |  |  |  |  |  |
| Swinnen SG, 2010 |  |  |  |  |  |  |  |
| Strojek K, 2010 |  |  |  |  |  |  |  |
| Fogelfeld L, 2010 |  |  |  |  |  |  |  |
| Raskin P, 2009 |  |  |  |  |  |  |  |
| Rosenstock J, 2008 |  |  |  |  |  |  |  |
| Hollander P, 2008 |  |  |  |  |  |  |  |
| Pan CY, 2007 |  |  |  |  |  |  |  |
| Yokoyama H, 2006 |  |  |  |  |  |  |  |
| Yki-Jarvinen H, 2006 |  |  |  |  |  |  |  |
| Hermansen K, 2006 |  |  |  |  |  |  |  |
| Eliaschewitz FG, 2006 |  |  |  |  |  |  |  |
| Haak T, 2005 |  |  |  |  |  |  |  |
| Riddle MC, 2003 |  |  |  |  |  |  |  |
| Massi Benedetti M, 2003 |  |  |  |  |  |  |  |
| Fritsche A, 2003 |  |  |  |  |  |  |  |
| Yki-Jarvinen H, 2000 |  |  |  |  |  |  |  |

Low Risk Some Concerns High
